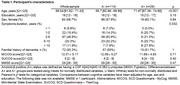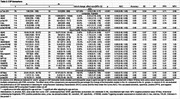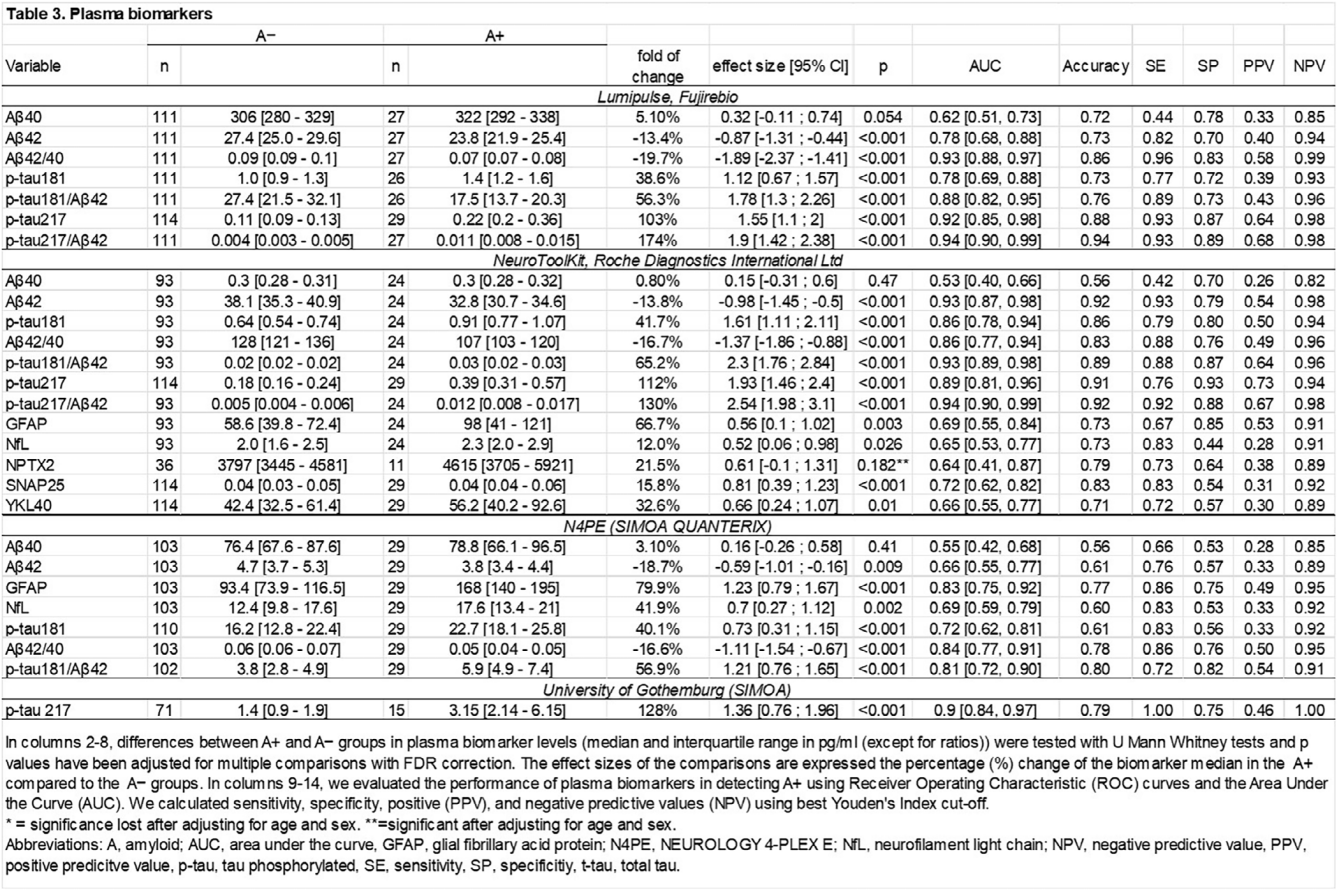# Detecting Amyloid–β Pathology in Subjective Cognitive Decline using novel CSF and plasma biomarkers

**DOI:** 10.1002/alz70856_105115

**Published:** 2026-01-07

**Authors:** José Contador, Federico Emanuele Pozzi, Gonzalo Sánchez‐Benavides, Ana Fernández‐Arcos, Carolina Minguillón, Karine Fauria, Armand González Escalante, Paula Ortiz‐Romero, Clara Quijano‐Rubio, Gwendlyn Kollmorgen, Nathalie Le Bastard, Alicia Nadal, Fernando Gonzalez‐Ortiz, Juan Lantero Rodriguez, Laia Montoliu‐Gaya, Eugeen Vanmechelen, Javier Torres‐Torronteras, Nicholas Ashton, Henrik Zetterberg, Kaj Blennow, Juan Domingo Gispert, Jose Luis Molinuevo, Marta del Campo, Oriol Grau‐Rivera, Marc Suárez‐Calvet

**Affiliations:** ^1^ Servei de Neurologia, Hospital del Mar, Barcelona, Spain; ^2^ Hospital del Mar Research Institute (IMIM), Barcelona, Spain; ^3^ Barcelonaβeta Brain Research Center (BBRC), Pasqual Maragall Foundation, Barcelona, Spain; ^4^ Fondazione IRCCS San Gerardo, Monza, Italy; ^5^ Milan Center for Neuroscience (NeuroMI), Milan, Italy; ^6^ Hospital del Mar Research Institute, Barcelona, Spain; ^7^ Fondazione IRCCS San Gerardo dei Tintori, Monza, Italy; ^8^ Centro de Investigación Biomédica en Red de Fragilidad y Envejecimiento Saludable (CIBERFES), Madrid, Spain; ^9^ Centro de Investigación Biomédica en Red de Fragilidad y Envejecimiento Saludable (CIBERFES), Instituto de Salud Carlos III, Madrid, Spain; ^10^ Department of Medicine and Life Sciences, Universitat Pompeu Fabra, Barcelona, Spain; ^11^ Roche Diagnostics International Ltd., Rotkreuz, Switzerland; ^12^ Roche Diagnostics GmbH, Penzberg, Germany; ^13^ Fujirebio Europe, Ghent, Belgium; ^14^ Fujirebio Iberia, Barcelona, Spain; ^15^ Institute of Neuroscience and Physiology, Department of Psychiatry and Neurochemistry, The Sahlgrenska Academy at University of Gothenburg, Mölndal, Sweden; ^16^ Clinical Neurochemistry Laboratory, Sahlgrenska University Hospital, Mölndal, Gothenburg, Sweden; ^17^ Clinical Neurochemistry Laboratory, Sahlgrenska University Hospital, Mölndal, Sweden; ^18^ ADx NeuroSciences NV, Technologiepark 94, Gent, Belgium; ^19^ Banner Sun Health Research Institute, Sun City, AZ, USA; ^20^ Institute of Neuroscienace and Physiology, University of Gothenburg, Mölndal, Västra Götaland, Sweden; ^21^ King's College London, Institute of Psychiatry, Psychology & Neuroscience, Maurice Wohl Clinical Neuroscience Institute, London, United Kingdom; ^22^ Banner Alzheimer's Institute, Phoenix, AZ, USA; ^23^ Hong Kong Center for Neurodegenerative Diseases, Hong Kong, Science Park, China; ^24^ Wisconsin Alzheimer's Disease Research Center, University of Wisconsin‐Madison, School of Medicine and Public Health, Madison, WI, USA; ^25^ Department of Neurodegenerative Disease, UCL Institute of Neurology, Queen Square, London, United Kingdom; ^26^ Department of Psychiatry and Neurochemistry, Institute of Neuroscience and Physiology, The Sahlgrenska Academy, University of Gothenburg, Mölndal, Sweden; ^27^ UK Dementia Research Institute at UCL, London, United Kingdom; ^28^ Clinical Neurochemistry Laboratory, Sahlgrenska University Hospital, Mölndal, Västra Götaland län, Sweden; ^29^ Paris Brain Institute, ICM, Pitié‐Salpêtrière Hospital, Sorbonne University, Paris, France; ^30^ Centro de Investigación Biomédica en Red de Fragilidad y Envejecimiento Saludable (CIBERFES), Instituto de Salud Carlos III, Barcelona, Spain; ^31^ Hospital del Mar Research Institute, Barcelona, Barcelona, Spain

## Abstract

**Background:**

Cerebrospinal fluid (CSF) and blood‐based biomarkers are essential tools for detecting individuals with cognitive complaints and at‐risk of Alzheimer's disease (AD). However, their utility in detecting underlying AD pathology in subjective cognitive decline (SCD) remains to be fully established, and direct comparisons between different biomarkers and platforms are needed. This study aimed to compare the performance of CSF and blood‐based biomarkers for detecting Amyloid‐b (Ab) pathology in individuals with SCD, including core AD and AD‐related pathophysiology biomarkers

**Method:**

We studied individuals with SCD from the β‐AARC cohort, which enrolled individuals seeking medical advice for cognitive complaints. Ab‐positivity (A+) was defined as CSF Aβ42/Aβ40<0.062 (Lumipulse). The following CSF biomarkers were compared: *p*‐tau181, t‐tau, α‐synuclein, GFAP, IL‐6, NfL, Neurogranin, S100B, sTREM2, YKL40, and tau variants (NTA‐tau, *p*‐tau205, *p*‐tau235, and *p*‐tau231). Plasma biomarkers included Aβ42/Aβ40, *p*‐tau181, *p*‐tau217, GFAP, NfL, NPTX2, SNAP25 and YKL40. Calculated ratios of amyloid and tau biomarkers in CSF and plasma were also analyzed. Biomarkers were analyzed using either Lumipulse, Simoa or Elecsys platforms. Diagnostic performance was assessed using Receiver Operating Characteristic (ROC) analysis, with sensitivity, specificity and negative (NPV) and positive predictive value (PPV) derived from the optimal Youden's Index cutoff.

**Result:**

We analyzed 143 individuals with SCD (25% A+; Table 1) Beyond AD CSF core biomarkers (Aβ42/Aβ40, *p*‐tau181), CSF *p*‐tau231, *p*‐tau205, and *p*‐tau235 showed the largest effect sizes among significant biomarkers (*p* <0.05, FDR corrected, Table 2), and achieved an NPV>0.95 for detecting amyloid status in CSF. In plasma (Table 3), *p*‐tau217 and *p*‐tau217/Aβ42 by either Lumipulse, Elecsys or Simoa, as well as *p*‐tau181/Aβ42 (Elecsys), and Aβ42/Aβ40 (Lumipulse) exhibited the highest discrimination accuracies (AUC >0.90, NPV >0.95). However, only CSF *p*‐tau181/Aβ42 (Lumipulse), showed a PPV>90%.

**Conclusion:**

Several CSF and plasma biomarkers distinguished A+ from A− individuals with cognitive complaints but normal cognition. Furthermore, plasma biomarkers are effective for ruling out Aβ pathology in SCD individuals, showing high NPVs. However, due to modest PPVs, their utility for confirmation is limited in low A+ prevalence settings like in the β‐AARC study.